# Differential expression of GABA_A_ receptor subunits δ and α6 mediates tonic inhibition in parvalbumin and somatostatin interneurons in the mouse hippocampus

**DOI:** 10.3389/fncel.2023.1146278

**Published:** 2023-07-20

**Authors:** Tzu-Hsuan Huang, Yi-Sian Lin, Chiao-Wan Hsiao, Liang-Yun Wang, Musa Iyiola Ajibola, Wahab Imam Abdulmajeed, Yu-Ling Lin, Yu-Jui Li, Cho-Yi Chen, Cheng-Chang Lien, Cheng-Di Chiu, Irene Han-Juo Cheng

**Affiliations:** ^1^Institute of Brain Science, National Yang Ming Chiao Tung University, Taipei, Taiwan; ^2^Institute of Biomedical Informatics, National Yang Ming Chiao Tung University, Taipei, Taiwan; ^3^Program in Genetics and Genomics, Baylor College of Medicine, Houston, TX, United States; ^4^Program in Molecular Medicine, National Yang Ming Chiao Tung University and Academia Sinica, Taipei, Taiwan; ^5^Institute of Neuroscience, National Yang Ming Chiao Tung University, Taipei, Taiwan; ^6^Taiwan International Graduate Program in Interdisciplinary Neuroscience, College of Life Sciences, National Yang Ming Chiao Tung University and Academia Sinica, Taipei, Taiwan; ^7^Department of Physiology, Faculty of Basic Medical Sciences, College of Health Sciences, University of Ilorin, Ilorin, Nigeria; ^8^Brain Research Center, National Yang Ming Chiao Tung University, Taipei, Taiwan; ^9^Department of Neurosurgery, China Medical University Hospital, Taichung, Taiwan; ^10^Spine Center, China Medical University Hospital, Taichung, Taiwan; ^11^Graduate Institute of Biomedical Science, China Medical University, Taichung, Taiwan; ^12^School of Medicine, China Medical University, Taichung, Taiwan

**Keywords:** tonic inhibition, somatostatin (SST), parvalbumin (PV), RiboTag, GABA_A_ receptor, GABRD, RNA-seq

## Abstract

Inhibitory γ-aminobutyric acid (GABA)-ergic interneurons mediate inhibition in neuronal circuitry and support normal brain function. Consequently, dysregulation of inhibition is implicated in various brain disorders. Parvalbumin (PV) and somatostatin (SST) interneurons, the two major types of GABAergic inhibitory interneurons in the hippocampus, exhibit distinct morpho-physiological properties and coordinate information processing and memory formation. However, the molecular mechanisms underlying the specialized properties of PV and SST interneurons remain unclear. This study aimed to compare the transcriptomic differences between these two classes of interneurons in the hippocampus using the ribosome tagging approach. The results revealed distinct expressions of genes such as voltage-gated ion channels and GABA_A_ receptor subunits between PV and SST interneurons. *Gabrd* and *Gabra6* were identified as contributors to the contrasting tonic GABAergic inhibition observed in PV and SST interneurons. Moreover, some of the differentially expressed genes were associated with schizophrenia and epilepsy. In conclusion, our results provide molecular insights into the distinct roles of PV and SST interneurons in health and disease.

## 1. Introduction

Excitatory and inhibitory neurons form the foundation of neuronal networks and coordinate neuronal activities under physiological conditions. Despite constituting only 10–20% of total neurons, local-circuit γ-aminobutyric acid (GABA) inhibitory neurons, also known as GABAergic interneurons, play vital roles in maintaining the balance of excitation-inhibition within neuronal circuits. Consequently, dysfunction of inhibition is associated with various brain disorders (Belelli et al., 2009; Baroncelli et al., 2011; Ruden et al., 2021; Song et al., 2021; Yang et al., 2022). GABAergic interneurons release GABA to regulate neuronal excitability through either phasic or tonic inhibition (Szabadics et al., 2007; Brickley and Mody, 2012). Phasic inhibition is mediated by synaptic GABA receptors, whereas tonic inhibition is generated by extrasynaptic GABA receptors (Farrant and Nusser, 2005). These two modes of inhibition are mediated by GABA_A_ receptors composed of different subunits (Prenosil et al., 2006; Lee and Maguire, 2014).

Parvalbumin (PV)- and somatostatin (SST)-expressing interneurons are the two major classes of GABAergic interneurons. They have distinct morphological features, synaptic connectivity, and physiological properties (Hu et al., 2014; Lee et al., 2016; Booker and Vida, 2018). Morphologically, PV interneurons, including basket cells and axon-axonic cells, selectively project their axons to perisomatic regions of target neurons. On the other hand, SST interneurons, which comprise multiple cell subtypes, preferentially target their dendritic domains (Lee et al., 2016; Booker and Vida, 2018). Therefore, PV interneurons selectively control spike generation by strategically positioning synapses near the axon initial segments, whereas SST interneurons regulate synaptic plasticity by tuning dendritic membrane potential (Miles et al., 1996; Klausberger and Somogyi, 2008; Kepecs and Fishell, 2014). Under physiological conditions, PV and SST interneurons coordinate and synchronize network activities through distinct spatial and temporal domains (Kepecs and Fishell, 2014). PV and SST interneurons fire at different phases during neuronal synchronization. For instance, PV interneurons fire preferentially at the descending phase of theta oscillations, whereas SST interneurons fire rhythmically at the trough phase of theta cycles (Klausberger et al., 2003). Moreover, PV interneurons promote the synchronization of spike times when instantaneous firing rates are low (<12 Hz), whereas SST interneurons preferentially promote the synchronization of spike times when instantaneous firing rates are high (>12 Hz) (Jang et al., 2020). PV interneurons exhibit characteristics of fast-spiking neurons, whereas SST interneurons display traits of non-fast-spiking neurons (Lee et al., 2016). PV interneurons are characterized by low input resistance, fast membrane time constant, and depressing excitatory inputs (Jonas et al., 2004; Hu et al., 2014). In contrast, SST interneurons possess high input resistance, slow membrane time constant, and facilitate excitatory inputs (Pouille and Scanziani, 2004). Thus, PV and SST interneurons manifest distinguishable spike probabilities upon repetitive excitation (Pouille and Scanziani, 2004; Kapfer et al., 2007; Silberberg and Markram, 2007; Silberberg, 2008). PV interneurons are primarily activated during the initial phase of excitation inputs, whereas SST interneurons are preferentially recruited during the late phase of repetitive excitation (Pouille and Scanziani, 2004).

γ-aminobutyric acid exerts powerful inhibition on neuronal excitability by activating fast ionotropic GABA_A_ and slow metabotropic GABA_B_ receptors. Stimulation of the GABA_B_ receptor causes a prolonged decrease in neuronal excitability via the inhibition of adenylyl cyclase and voltage-gated Ca^2+^ channels as well as the opening of G protein-coupled inward-rectifying potassium channels (Mannoury la Cour et al., 2008). GABA_A_ receptors are heteropentameric chloride channels assembled by various combinations of 19 subunits (Sigel and Steinmann, 2012). Different subunit compositions determine their electrophysiological properties, cell surface distribution, and pharmacological response (Farrant and Nusser, 2005). The δ subunit is one of the most relevant subunits that mediate a slow constant inhibitory current called tonic inhibition and is expressed in most brain regions (Arslan, 2021; Sun et al., 2022). The human transcriptomic dataset from the Allen Brain Institute indicates that the expression of the δ subunit of GABA_A_R (*Gabrd*) in PV interneurons is higher than that in other interneurons, such as SST and vasoactive intestinal polypeptide interneurons (Field et al., 2021). Thus, tonic inhibition can differentially modulate the excitability of neuron subtypes (Glykys et al., 2008; Lee and Maguire, 2014; Bryson et al., 2020).

Gene expression dictates cellular functions. Recent advances in cell type-specific gene profiling techniques have yielded valuable insights into the physiological functions of a cell type. Conventionally, microfluidic or magnetic bead-based cell sorting is used to isolate the defined cell type from dissociated tissues (Haimon et al., 2018). Owing to the morphological complexity and relatively low abundance of GABAergic inhibitory interneurons, these standard cell dissociation and isolation protocols may not be as effective in capturing the mRNA of inhibitory interneurons. Moreover, extensive cell isolation procedures can damage mature neurons and potentially modify gene expression. Cell type-specific ribosome tagging (RiboTag) followed by RNA sequencing (RNA-seq) provides an alternative method to analyze the transcriptome of sparse cells in mouse brains without disruptive cell isolation.

Previous studies have applied RiboTag to cortical inhibitory interneurons (Mardinly et al., 2016; Huntley et al., 2020). This study aimed to understand the functional differences between PV and SST cells in the mouse hippocampus. To achieve this, we employed the RiboTag method to isolate cell type-specific mRNA transcripts *in vivo*. Subsequently, RNA sequencing was performed to compare the differentially expressed genes between PV and SST interneurons in the hippocampus. To validate the significance of these findings, potential genes and pathways of interest were confirmed through additional techniques such as real-time quantitative polymerase chain reaction (RT-qPCR), immunofluorescence staining, and electrophysiology. Our findings indicate that the upregulation of *Gabrd* and *Gabra6* potentially contributes to the contrasting tonic GABAergic inhibition observed between PV and SST interneurons. Moreover, some differentially expressed genes identified in our analysis have been linked to conditions such as schizophrenia and epilepsy.

## 2. Materials and methods

### 2.1. Animals

**FIGURE 1:**
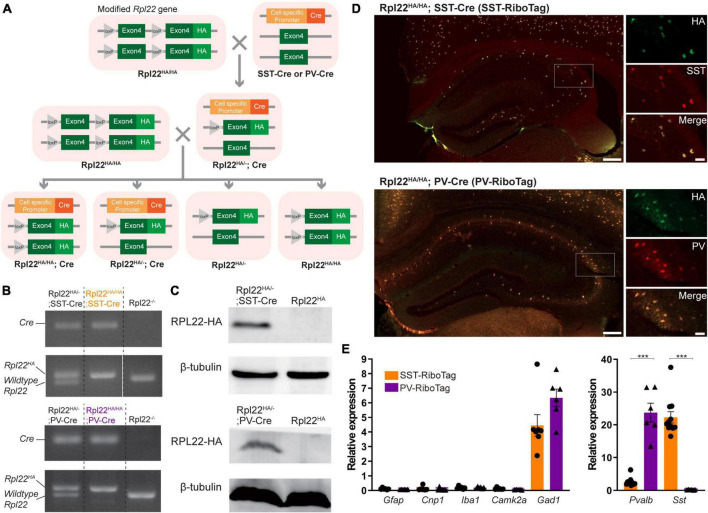
Cell type-specific expressions of Rpl22-HA (RiboTag) in hippocampal SST and PV neurons. **(A)** The breeding strategy for generating Rpl22^HA/HA^; PV-Cre (PV-RiboTag) or Rpl22^HA/HA^; SST-Cre (SST-RiboTag) mice. **(B)** Representative PCR genotyping images with primers recognizing Cre and Rpl22 alleles. **(C)** Immunoblotting using HA antibody indicating Rpl22-HA expression in the hippocampus of PV-RiboTag or SST-RiboTag mice. **(D)** Representative immunofluorescence images demonstrate that HA-expressing cells (green) are colocalized with either SST or PV cells (red). The scale bars represent 200 (left) μm and 50 (right) μm. **(E)** RT-qPCR measurement of mRNA levels of markers for astrocyte (*Gfap*) (SST-RiboTag, *n* = 6; Rpl22^HA^; PV-RiboTag, *n* = 6), oligodendrocytes (*Cnp1*) (SST-RiboTag, *n* = 7; PV-RiboTag, *n* = 7), microglia (*Iba1*) (SST-RiboTag, *n* = 6; PV-RiboTag, *n* = 6), pyramidal neuron (*Camk2a*) (SST-RiboTag, *n* = 6; PV-RiboTag, *n* = 5), GABAergic inhibitory interneuron (*Gad1*) (SST-RiboTag, *n* = 7; PV-RiboTag, *n* = 6), PV cell (*Pvalb*) (SST-RiboTag, *n* = 7; PV-RiboTag, *n* = 6), and SST cell (*Sst*) (SST-RiboTag, *n* = 11; PV-RiboTag, *n* = 6) in ribosome-bound mRNA isolated from PV-RiboTag (purple) and SST-RiboTag mice (orange). Data are presented as the mean ± SEM and analyzed using the Mann–Whitney test. ***p < 0.001. The detailed mice age, gender and the statistical analysis results were listed in Supplementary Table 3.

For cell-type specific expression of HA-tagged ribosomes in PV or SST interneurons, the RiboTag mice (B6N.129-*Rpl22^tm1.1Psam^*/J, JAX^®^ Strain # 011029) were crossed with either PV-Cre driver mice (B6;129P2-*Pvalb^tm1(cre)Arbr^*/J, JAX^®^ Strain # 008069) or SST-Cre driver mice (B6N.Cg-*Sst^tm2.1(cre)Zjh^*/J, JAX^®^ Strain # 13044) (Figure 1A). The RiboTag mouse line carries an Rpl22 allele fused to Cre-induced hemagglutinin (HA) (RPL22^HA^) that facilitates the isolation of ribosome-bound mRNA specifically from Cre + cells by immunoprecipitation (Sanz et al., 2009). For electrophysiology recording, the Ai14 reporter mice [B6.Cg-Gt(ROSA)26Sor*^tm14(CAG–tdTomato)Hze^*/J, JAX^®^ Strain # 007914] were crossed with either PV-Cre or SST-Cre driver mice. Mice were housed in groups of 2–5 in a standard cage under a 12-h light-dark cycle at 25°C and 60% humidity and provided food and water *ad libitum*. The detailed information for mice used in each experiment is listed in Supplementary Table 3. This study was approved by the Institutional Animal Care and Use Committee (IACUC) of the National Yang Ming Chiao Tung University. This study followed all applicable international, national, and institutional guidelines for the care and use of animals.

### 2.2. Genotyping

A 2-mm mouse tail was cut and incubated with 50 μl 1X DNA isolation buffer (25 mM NaOH and 0.2 mM EDTA) at 98°C for 1 h. The lysate was neutralized with 50 μl 40 mM Tris-HCl (pH 5.5) and spun down. Then, 1 μl of the supernatant was taken as the DNA template for PCR amplification by using a PCR dye master mix (ADPMX02D-100; Arrowtech, USA or SA-PB10.44-05; PCR Biosystems, USA) according to the manufacturer’s instructions. The PV-Cre allele was amplified with the forward primer 5′-CAGAGCAGGCATGGTGACTA-3′ and reverse primer 5′-AGTACCAAGCAGGCAGGAGA-3′. The SST-Cre allele was amplified with the forward primer 5′-TGGTTTGTCCAAACTC ATCAA-3′ and reverse primer 5′-GGGCCAGGAGTTAAGG AAGA-3′. The Rpl22^HA^ allele was amplified with the forward primer 5′-GGGAGGCTTGCTGGATATG-3′ and the reverse primer 5′-TTTCCAGACACAGGCTAAGTACAC-3′. PCR was performed under the touchdown cycling protocol suggested by the Jackson Laboratory. All PCR products were analyzed by electrophoresis on 3% agarose gel. The size of the PCR product was 100 bp for the PV-Cre allele, 200 bp for the SST-Cre allele, 260 bp for the wild-type Rpl22 allele, and 290 bp for the Rpl22^HA^ allele.

### 2.3. HA-tagged ribosome immunoprecipitation and RNA extraction

Mice were executed by cervical dislocation without euthanasia. The hippocampi from either Rpl22^HA^;PV-Cre (PV-RiboTag) or Rpl22^HA^;SST-Cre (SST-RiboTag) mice were dissected and homogenized in 500 μl polysome buffer (50 mM Tris-HCl, pH 7.4, 12 mM MgCl_2_, 100 mM KCl, 1 mM DTT, 1% NP40, 200 U/ml ribonuclease inhibitor, 0.5 mg/ml heparin, 100 μg/ml cycloheximide, and 10 μl/ml Protease inhibitor cocktail) using a bead-based tissue homogenizer (Bullet Blender^®^; Next Advance, Inc.). The lysate was centrifuged at 4°C at 16,000 × *g* for 10 min, and 30 μl of supernatant was collected as an input for validation. The remaining supernatant was incubated with the anti-HA antibody at 4°C for 4 h with rotary agitation. Pre-washed protein G beads were added to the antibody homogenate and incubated overnight at 4°C with rotary agitation. After 12 h, the samples were washed three times with high-salt buffer (50 mM Tris-HCl, pH 7.4, 12 mM MgCl_2_, 300 mM KCl, 1 mM DTT, 1% NP40, 100 U/ml Ribonuclease inhibitor, 100 μg/ml cycloheximide, 5 μl/ml protease inhibitor cocktail). Lysis buffer (Qiagen RLT buffer containing 2-Mercaptoethanol) was added to the beads and input samples. Total RNA was purified using the Qiagen RNeasy Micro kit (Cat. No. 74004; Qiagen) according to the manufacturer’s instructions and quantified with a Qubit fluorometer. Information for all chemicals and antibodies is listed in Supplementary Tables 1, 2.

### 2.4. Immunoblotting

The immunoprecipitation (IP) and input samples were incubated with SDS sample buffer (87.5 mM Tris-HCl, 1% SDS, 30% glycerol, 0.6 M DTT, and 180 μM bromophenol blue, pH 6.8) at 95°C for 10 min. Proteins were separated by 15% Tris-glycine SDS-polyacrylamide gel electrophoresis and transferred to nitrocellulose membranes. After blocking in casein blocking buffer, the membranes were probed with rabbit anti-HA (1:1000; ab9110; Abcam) and mouse anti-beta tubulin (1:2000; ab7751; Abcam) antibodies. The membranes were washed in Tris-buffered saline Tween and probed with horseradish peroxidase-conjugated anti-mouse IgG (1:1000; Jackson ImmunoResearch) and anti-rabbit IgG Trueblot^®^ (1:1000; Rockland). Proteins signals were developed by using a chemiluminescent substrate ECL detection system (Millipore) and imaged with a luminescence camera system (LAS4000; Fujifilm). Information for all chemicals and antibodies is listed in Supplementary Tables 1, 2.

### 2.5. Immunofluorescence staining

Somatostatin-RiboTag mice and PV-RiboTag mice were euthanized by intraperitoneal administration of urethane (1,500 mg/kg), followed by perfusion with normal saline and 4% paraformaldehyde through the myocardial vascular system. The brains were fixed in 4% paraformaldehyde/PBS for 20 h and then embedded with 30% sucrose overnight. The embedded brains were frozen in an optimal cutting temperature medium (Tissue-Tek^®^ O.C.T. Compound, 4583, SAKURA) and sliced coronally at a 30-μm thickness in −20°C Cryostat (Thermo Cryostar NX70). The brain slices were washed three times with 1X PBS, permeabilized in 0.5% Triton X-100/PBS, and blocked with 10% blocking buffer (0.1% Triton X-100, 10% fetal bovine serum, 3% bovine serum albumin, and 0.1% sodium azide in PBS). The slices were then incubated with primary antibodies against HA (1:1000; GTX115044; GeneTex or 1:200; MMS-101R; Biolegend), GABRD (1:200; PA5-77408; Invitrogen), GABRA6 (1:200; NB300-196; Novus) SST (1:100; MAB354; Millipore), and PV (1:1000; MAB1572; Millipore) overnight at 4°C; washed three times with 1X PBS. Afterward, they were washed three times with 1X PBS, followed by a 2-h incubation at room temperature with the respective secondary antibodies: anti-rabbit Alexa 488 (1:500), anti-rabbit Alexa 594 (1:500), anti-mouse Alexa 488 (1:500), anti-mouse Alexa 594 (1:500) and anti-rat Alexa 594 (1:1000). Finally, the slices were mounted using VECTASHIELD^®^ mounting medium (Vector Laboratories). Images were captured by fluorescence microscopy (Olympus BX63 and Zeiss Apotome.2); 120 HA-positive cells in the hippocampus were enrolled in 1 mouse sample, and the average integration ratio of GABRD or GABRA6 overlap HA signals were quantified by MetaMorph Premier analysis software. Information for chemicals and antibodies is listed in Supplementary Tables 1, 2.

### 2.6. RT-qPCR

The purified RNA was generated into cDNA using an oligo-dT primer and SuperScript II reverse transcriptase (ERT12925K; Lucigen). The level of specific mRNA was analyzed using specific primer pairs (listed in Supplementary Table 4). Samples were mixed with 2X qPCRBIO SyGreen Blue Mix HI-ROX (PB20.16-01, PCR Biosystems) and analyzed on the StepOnePlus real-time PCR system. Quantitative polymerase chain reaction (qPCR) was carried out under the following conditions: an initial denaturation step at 95°C for 3 min, 40 cycles of denaturation at 95°C for 30 s, and annealing/extension at 60°C for 30 s. The *Gapdh* gene was used as an internal control. Normalized mRNA levels were quantified using the 2^––ΔΔCt^ method.

### 2.7. RNA sequencing

The purified RNA samples were treated with DNase I to remove DNA contamination before subjecting for library preparation. The RNA quality was tested using the Agilent 2100 Bioanalyzer (Agilent Technologies, Inc., Santa Clara, CA, USA). Samples with an RNA integrity number (RIN) greater than 6.6 were subjected to RNA sequencing library preparation. For samples with total RNA < 10 ng, the RNA sequencing libraries were prepared using the Switching Mechanism At the 5′ end of RNA Template (SMART)-Seq Stranded Kit (Takara Bio USA, Inc., San Jose, CA, USA), which incorporated SMART cDNA synthesis technology and preserved the strand orientation of the original RNA. For samples with total RNA > 100 ng, the RNA sequencing libraries were prepared using Illumina TruSeq Stranded mRNA Sample Preparation Kit (Illumina Inc., USA) which produced directional RNA-seq libraries. The RNA libraries were quantified by qPCR, and the quality was assessed with the 2100 Bioanalyzer HS DNA Kit. The RNA libraries were sequenced on a NextSeq550 (Illumina, Inc., San Diego, CA, USA) by paired-end sequencing with a 75-bp read length to a minimum depth of 30–70 million reads. The low quality of bases (<Q20), the first 12 bases, and adapters were trimmed from the dataset. The reads were mapped to Genome Reference Consortium Mouse Build 38 (GRCm38/mm10) and run RNA-seq analysis by CLC Genomics Workbench (QIAGEN, Germany). Expression levels were measured in transcripts per million (TPM), and the expression level for a gene was calculated as the sum of the TPM values of its isoforms.

### 2.8. Differential expression analysis

Differential gene expression was analyzed using DESeq2 v1.32.0 (Love et al., 2014). Genes with low counts (sum < 10 in each sample) were filtered out. The design formula was to compare the difference in gene expression between two cell types while controlling for batch effect. The cutoff for differentially expressed genes (DEGs) was |log2foldchange| > 1 and an adjusted *P* < 0.05 (Benjamini-Hochberg method). Results were visualized with a volcano plot generated using the EnhancedVolcano v1.10.0^1^ and Pretty Heatmap (pheatmap v1.0.12).

### 2.9. Functional enrichment analysis

Differentially expressed genes were subjected to functional enrichment characterization with the biological process (BP) of Gene Ontology (GO) (Release 2021-09-01) and pathways in the Kyoto Encyclopedia of Genes and Genomes (KEGG) (Release 100.0) using clusterProfiler v4.0.5 (Wu et al., 2021). Over-representation tests were used to identify significant GO terms and KEGG pathways, and the significance threshold was set at a *P*-value of <0.05. Results were visualized with treeplots using the Enrichplot v1.12.3. The Ward.D method was used to cluster enriched terms. A set of succinct representative words were manually assigned for each cluster.

### 2.10. Disease gene sets

Enriched schizophrenia and epilepsy-related gene sets were extracted from the web server “Enrichr” (Chen et al., 2013; Kuleshov et al., 2016) using DEGs as input (results downloaded on Oct 6, 2021). Only overlapping DEGs were shown on the heatmap.

### 2.11. Virus and stereotaxic surgery

To specifically label PV neurons in the dentate gyrus (DG), we injected AAV5-hSyn-DIO-mCherry or AAV1-hDlx-DIO-tdtomato virus into PV-Cre mice. In the stereotaxic surgery, mice were deeply anesthetized with isoflurane (4% induction, 1.5–2% maintenance in O_2_; Halocarbon Laboratories, North Augusta, SC, USA) and placed in a stereotaxic injection frame (IVM-3000; Scientifica, Uckfield, UK). During all surgical procedures, mice were kept on a heating pad (Physiological Biological Temperature Controller TMP-5b, Supertech Instruments, Budapest, Hungary) to maintain their surface body temperatures at 34°C. After securing the head with ear bars, 75% ethanol was used to sterilize the surgical area, and the eyes were protected using an ophthalmic gel. The injections were performed using the following stereotaxic coordinates: 3.5 mm posterior from bregma, 2.8 mm lateral from the midline on both sides, 3 and 3.2 mm ventral from the cortical surface. For viral injections, we bilaterally injected 0.3 μL of the viral solution into each location, using a 10-μL NanoFil syringe and a 34-G beveled metal needle (World Precision Instruments, Sarasota, FL, USA). The flow rate (0.1 μL/min) was controlled with a nanopump controller (KD Scientific, Holliston, MA, USA). After viral injection, the needle was raised 0.1 mm above the injection site for an additional 10 min to allow the virus to diffuse before being withdrawn slowly. After withdrawing the needle, the incision was closed by suturing, and the mice were returned to their home cage for recovery.

### 2.12. Brain slice preparation for electrophysiology

Transverse acute brain slices (300-μm thick) containing the hippocampus were cut from SST-Cre; Ai14, PV-Cre; Ai14 mice using a vibratome (DTK-1000; Dosaka, Kyoto, Japan). The mice were anesthetized using isoflurane and rapidly decapitated. The brains were quickly removed and transferred to an ice-cold oxygenated (95% O_2_ and 5% CO_2_) sucrose cutting solution containing (in mM): 87 NaCl, 25 NaHCO_3_, 1.25 NaH2PO_4_, 2.5 KCl, 10 glucose, 75 sucrose, 0.5 CaCl_2_, and 7 MgCl_2_. After sectioning, the slices were recovered at 34°C for 30 min in a holding chamber filled with an oxygenated sucrose solution and then transferred to room temperature (22–24°C) for further experiments.

### 2.13. Patch clamp recording

For the whole-cell recordings, individual slices were transferred to a submerged chamber and were continuously perfused with oxygenated artificial cerebrospinal fluid (ACSF) containing the following (in mM): 125 NaCl, 25 NaHCO_3_, 1.25 NaH_2_PO_4_, 2.5 KCl, 25 glucose, 2 CaCl_2_, and 1 MgCl_2_. The tdTomato or mCherry expressing cells in the DG were visualized in the brain slices using epifluorescence. Then, the cells were recorded under an infrared differential interference contrast or infrared Dodt gradient contrast microscope (IR-DIC or IR-DGC, BX51WI, Olympus) equipped with an LED source (590 nm, LED4D162, controlled by DC4104 driver, Thorlabs, NJ, USA). Whole-cell patch-clamp recordings were performed at 22–24°C using Axopatch 200B amplifier or Multiclamp 700B amplifier (Molecular Devices, Sunnyvale, CA, USA). The recording electrode pipettes (2–7 MΩ) pulled from borosilicate glass tubing (outer diameter, 1.5 mm; inner diameter, 0.86 mm; Harvard Apparatus) were filled with a high-Cl^–^ internal solution containing the following (in mM): 15 K-gluconate, 140 KCl, 0.1 EGTA, 2 MgCl_2_, 4 Na_2_ATP, 10 HEPES, 0.5 Na_3_GTP, and 0.4% biocytin (w/v, Life Technologies, Grand Island, NY, USA). GABA_A_R-mediated currents were blocked with the GABA type A receptor (GABA_A_R) antagonist SR95531 (10 μM), while δ-GABA_A_R-mediated currents were induced with the δ-GABA type A receptor (δ-GABA_A_R) agonist THIP (10 μM, MedChemExpress). Phasic and tonic GABA currents were recorded using a high Cl^–^ internal solution at a holding potential of −70 mV in voltage-clamp in the presence of kynurenic acid (Kyn, 2 mM), a blocker for ionotropic glutamatergic receptors.

Data were analyzed using Clampfit 10.3 (Molecular Devices, CA, USA). The amplitude of tonic inhibition was analyzed as the difference between the holding currents measured before and after the application of either the GABA_A_R antagonist SR95531 (10 μM) or the δ-GABA_A_R agonist THIP (10 μM). The holding current was calculated from average values of the 5-s epoch, without obvious spontaneous synaptic events, taken in three different segments before and after bath application of either 10 μM SR95531 or 10 μM THIP as previously described (Song et al., 2011; Gupta et al., 2012). Briefly, the magnitude of tonic GABA current was calculated by plotting all-point histograms of relevant 5-s segments of data. These data were fit to Gaussian equations, constraining fits to values two bins more negative than the peak. This ensured that the tail of higher-amplitude values representing spontaneous inhibitory postsynaptic currents (sIPSCs) did not influence the fit (Santhakumar et al., 2006, 2010). The peak value was designated as the average value of the holding currents. The current density was calculated by dividing the tonic GABA current by cell capacitance. The input resistance was measured by the ratio of a steady-state voltage response (last 100 ms of a 1-s pulse) versus a 10 pA hyperpolarizing current pulse injected (Liu et al., 2014; Ajibola et al., 2021). The signals were recorded using Multiclamp 700B amplifiers (Molecular Devices); filtered at 4 kHz; and sampled at 10 kHz using a digitizer (Digidata 1440A, Molecular Devices), which was controlled using the pCLAMP version_10.3 (Molecular Devices).

### 2.14. Statistical analysis

The differences in qPCR and immunofluorescence staining experiments between SST-RiboTag and PV-RiboTag mice were presented as the mean ± SEM and analyzed using the Mann-Whitney test. The detailed statistical results were listed in Supplementary Table 3. For RNA-seq analysis, DESeq2 (v1.32.0) was used with SST-RiboTag *n* = 5 and PV-RiboTag *n* = 7. Raw read counts were normalized with library size. Normalized counts were compared pairwise between groups and analyzed by Wald test. B-H adjusted *p*-value and log2 fold change were used to determine DEGs. Please see Supplementary Tables 5–9 for the Bioinformatic analysis statistics.

## 3. Results

### 3.1. Ribosome tagging isolates mRNA from PV and SST interneurons

Parvalbumin and SST interneurons are physiologically and anatomically distinct populations. To compare their differences at the molecular level, we employed the RiboTag approach to determine the transcriptome of PV and SST interneurons in the mouse hippocampus. The RiboTag mice were crossed with either PV-Cre or SST-Cre mice to drive the cell-type specific expression of HA-tagged ribosomes in either PV or SST interneurons (Figure 1A). The genotypes of Rpl22^HA/HA^; SST-Cre and Rpl22^HA/HA^; PV-Cre mice as determined by PCR are shown in Figure 1B. In the hippocampus, RPL22-HA protein was expressed only in the presence of Cre, as indicated by immunoblotting results (Figure 1C). The cell type-specific expression of HA-tagged ribosomes was confirmed by double immunoreactivities against HA with either PV or SST. All mice used in this study were Rpl22^HA/HA^ and indicated as SST-RiboTag and PV-RiboTag in the following text.

Hemagglutinin tag was only presented in either PV or SST interneurons in the hippocampus of SST-RiboTag or PV-RiboTag mice, respectively (Figure 1D). To check the enrichment of mRNAs from PV or SST interneurons, HA-tagged ribosomes were immunoprecipitated with an anti-HA antibody, and the ribosome-bound mRNAs were purified. Ribosome-bound mRNAs from both genotypes contained markers for GABAergic inhibitory interneurons (*Gad1*), and not all types of glia cells (*Gfap*, *Cnp1*, *Iba1*) and pyramidal neurons (*Camk2a*) (Figure 1E). *Sst* or *Pvalb* are the markers for these two types of interneurons. In ribosome-bound RNAs, *Sst* and *Pvalb* were highly enriched in SST- and PV-RiboTag mice, respectively (Figure 1E). These results indicated the specificity of the RiboTag approach to isolate mRNA from PV or SST inhibitory interneurons.

### 3.2. Voltage-gated ion channels are differentially expressed between PV and SST interneurons

**FIGURE 2:**
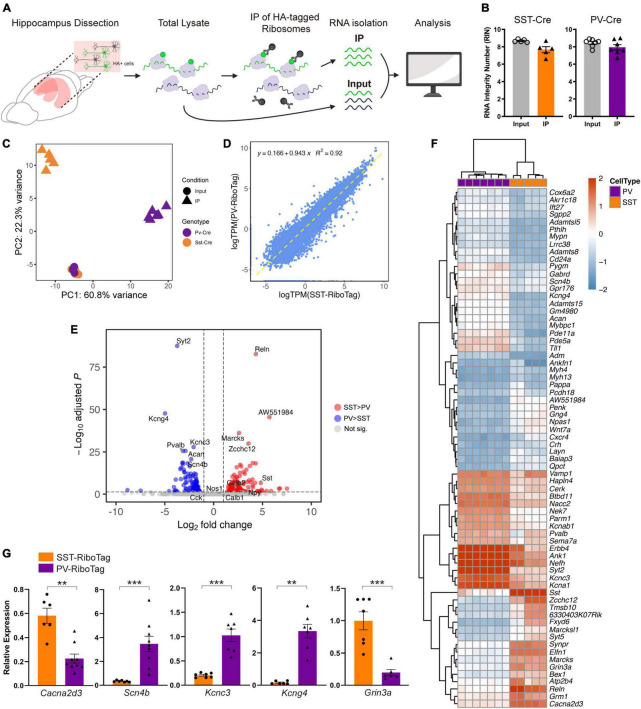
Differential gene expression profiles of PV and SST interneurons. **(A)** Workflow for the isolation of total RNA and cell type-specific RNA in the hippocampus. **(B)** RNA integrity number (RIN) of input and IP samples of PV-RiboTag (purple) and SST-RiboTag mice (orange). **(C)** Principal component analysis of 10 SST-Cre samples (5 IP-input pairs) and 14 PV-Cre samples (7 IP-input pairs). **(D)** Correlation between expression (TPM) of SST-IP and of PV-IP. **(E)** The volcano plot shows DEGs between SST and PV interneurons. Red dots indicate genes with significantly higher expression in SST interneurons than in PV interneurons. Blue dots represent genes with significantly higher expression in PV interneurons than in SST interneurons. Top DEGs (adjusted *P* < 10^–20^) and known markers for SST or PV interneurons are labeled. **(F)** Heatmap showing the top 70 DEGs between SST and PV interneurons ranked by adjusted *P*. **(G)** RT-qPCR quantification of mRNA expression of markers for selected DEGs: *Kcnc3* (SST-RiboTag, *n* = 7; PV-RiboTag, *n* = 7), *Kcng4* (SST-RiboTag, *n* = 6; PV-RiboTag, *n* = 7), *Cacna2d3* (SST-RiboTag, *n* = 6; PV-RiboTag, *n* = 9), *Scn4b* (SST-RiboTag, *n* = 7; PV-RiboTag, *n* = 9), *Grin3a* (SST-RiboTag, *n* = 7; PV-RiboTag, *n* = 6) in IP samples of PV-RiboTag (purple) and SST-RiboTag mice (orange). Data are presented as the mean ± SEM and analyzed using the Mann–Whitney test. ***p < 0.001, **p < 0.01. The detailed mice age, gender and the statistical analysis results were listed in Supplementary Table 3.

To profile cell type-specific gene expression, we purified the total mRNA (input) and immunoprecipitated HA-tagged ribosome-bound mRNA (IP) from SST-RiboTag or PV-RiboTag mice (Figure 2A). The RIN of samples used for RNA-seq ranged from 6.6 to 9.0 (Figure 2B). The principal component analysis (PCA) plot of mRNA profiles revealed three clusters from SST-RiboTag IP samples (orange ▲), PV-RiboTag IP samples (purple ▲), and input samples from both genotypes (○). The inputs from SST-RiboTag mice were undisguisable from those from PV-RiboTag mice (Figure 2C). Although mRNA from SST and PV interneurons can be separated on the PCA plot, its expression correlation was high (*R*^2^ = 0.92). A few points were distributed above or below the diagonal line, indicating that genes were expressed differentially between these two cell types (Figure 2D). In differential expression analysis, 106 DEGs were upregulated in SST interneurons, whereas 105 DEGs were upregulated in PV interneurons (|log2FoldChange| > 1; adjusted-*P* < 0.05) (Supplementary Table 5).

Among the upregulated genes in SST interneurons, *Reln* and *AW551984* showed the most significant differential expression. For genes that were upregulated in PV interneurons, *Syt2*, a marker for a subgroup of PV-expressing basket cells in the mouse hippocampus (Garcia-Junco-Clemente et al., 2010), reached the most significant expression level (Figure 2E). The top 70 most significant DEGs between SST and PV interneurons are highlighted in Figure 2F. SST interneurons were enriched with genes encoding auxiliary subunits of voltage-gated calcium channels (VGCCs), including *Cacna2d3*, *Cacng4*, and *Cacng5*. In contrast, the voltage-gated sodium channel gene *Scn4b* and a set of voltage-gated potassium channel genes (*Kcng4*, *Kcnab1*, *Kcnc3*, and *Kcna1*) were preferentially expressed in PV interneurons. An independent group of mice was used for RT-qPCR to confirm the RNA-Seq results. Genes that encode voltage-gated ion channels (*Cacna2d3*, *Scn4b*, *Kcnc3*, *Kcng4*, and *Grin3a*) all have significant differences between PV and SST interneurons (Figure 2G).

### 3.3. DEGs between PV and SST interneurons are enriched in synaptic transmission functions

**FIGURE 3:**
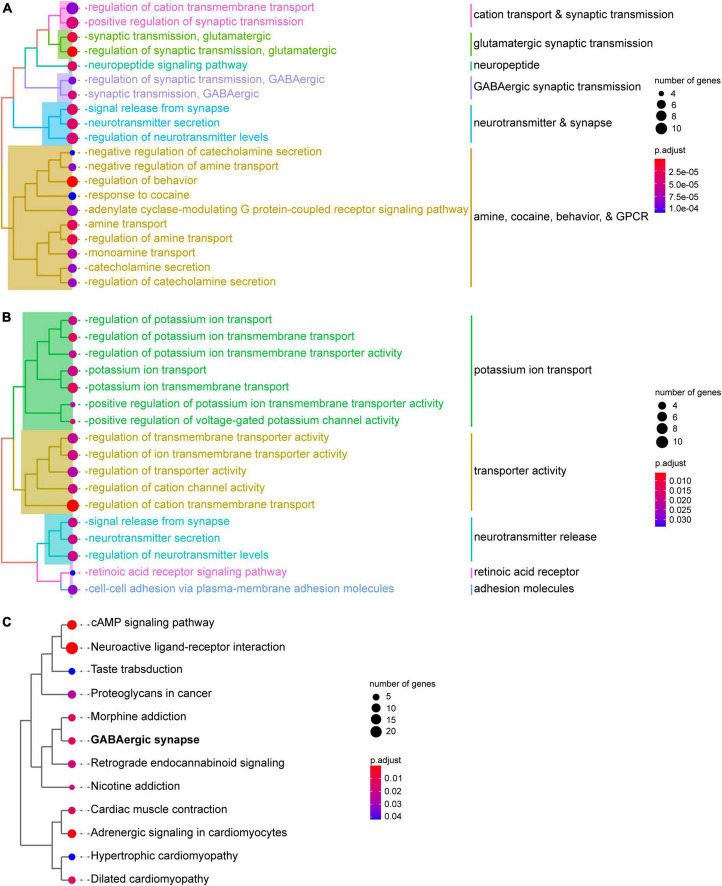
Functional enrichment analysis of PV and SST interneurons. **(A)** GO biological process enrichment shows the functions of genes with significantly higher expression in SST interneurons than in PV interneurons. **(B)** GO biological process enrichment shows functions of with significantly higher expression in PV interneurons than in SST interneurons. **(C)** KEGG pathway enrichment analysis of DEGs between SST and PV interneurons. GO terms or KEGG pathways are clustered using the ward.D method. The size of the circle represents the number of hit DEGs in each gene set. The color bar indicates the significance of enrichment.

To gain insight into the biological processes involved in SST and PV interneurons, we performed GO enrichment analyses for the DEGs. Genes with significantly higher expression in SST interneurons were enriched in GO categories of synaptic transmission, both glutamatergic and GABAergic. In addition, several clusters of genes related to amine-, cocaine-, behavior-, and G-protein-coupled receptor-related processes were also upregulated in SST interneurons (Figure 3A; Supplementary Table 6). Meanwhile, genes with significantly higher expression in PV interneurons were enriched in GO categories associated with potassium ion transport, transporter activity, and neurotransmitter release (Figure 3B; Supplementary Table 7). KEGG pathway enrichment analysis showed that the DEGs between SST and PV interneurons, regardless of direction, were enriched in neuroactive ligand-receptor interaction, drug addiction, GABAergic synapse, and several cardio-related pathways (Figure 3C; Supplementary Table 8).

### 3.4. GABA_A_ receptor genes are differentially expressed between PV and SST interneurons

**FIGURE 4:**
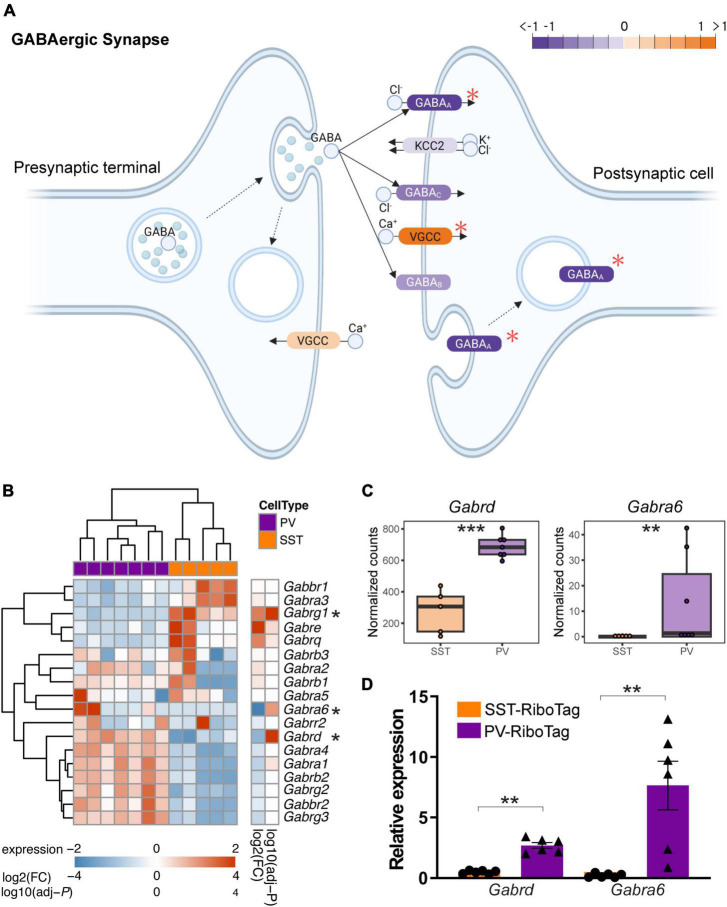
The difference in GABA receptor gene expression between PV and SST interneurons. **(A)** Modified KEGG Pathview showing the expression of SST and PV interneurons in the KEGG pathway “GABAergic synapse.” The orange indicates higher expression in SST interneurons, and the purple indicates higher expression in PV interneurons. The red asterisk indicates the gene with significant differential expression between SST and PV interneurons. **(B)** Heatmap showing the expression of GABA receptor-encoding genes in SST and PV interneurons. The asterisk indicates significant differential expression between SST and PV interneurons. The color bar shows variance stabilizing transformed expression, log2(fold change), and log10(adjusted *P*). **(C)** Boxplot showing the expression (normalized counts) of *Gabrd* and *Gabra6* in SST and PV interneurons in RNA-seq data. ***adjusted *P* < 0.001, **adjusted *P* < 0.01 in differential expression analysis. **(D)** RT-qPCR quantification of mRNA expression of markers for *Gabrd* and *Gabra6* in independent IP samples of PV-RiboTag (purple, *n* = 6) and SST-RiboTag mice (orange, *n* = 6). Data are presented as the mean ± SEM and analyzed using the Mann–Whitney test. **p < 0.01.

Synaptic dynamics are different between PV interneuron-principal neuron and SST interneuron-principal neuron connections (Klausberger and Somogyi, 2008; Kepecs and Fishell, 2014; Liu et al., 2014). Compared to interneuron-principal synapses, less is known about interneuron-interneuron synapses. Here, we further depicted the differential expression of the GABAergic synapse pathway in SST and PV interneurons in the KEGG (Figure 4A). Notably, GABA_A_-, GABA_B_-, and GABA_C_-receptors encoding genes that showed higher expression in PV interneurons outnumbered those in SST interneurons. In contrast, the genes expression for VGCCs were greater in SST interneurons than in PV interneurons. A heatmap for gene expression of GABA receptor subunits in SST and PV interneurons is shown in Figure 4B. Among these subunits, *Gabrd* and *Gabra6* both encoded GABA_A_ receptor subunits. RNA-seq data showed that the mRNA levels of both *Gabrd* and *Gabra6* were significantly higher in PV interneurons than in SST interneurons (Figure 4C). Increases in these two mRNAs were confirmed in the RT-qPCR analysis from the independent group of animals (Figure 4D).

### 3.5. PV interneurons exhibit larger tonic inhibition than do SST interneurons

**FIGURE 5:**
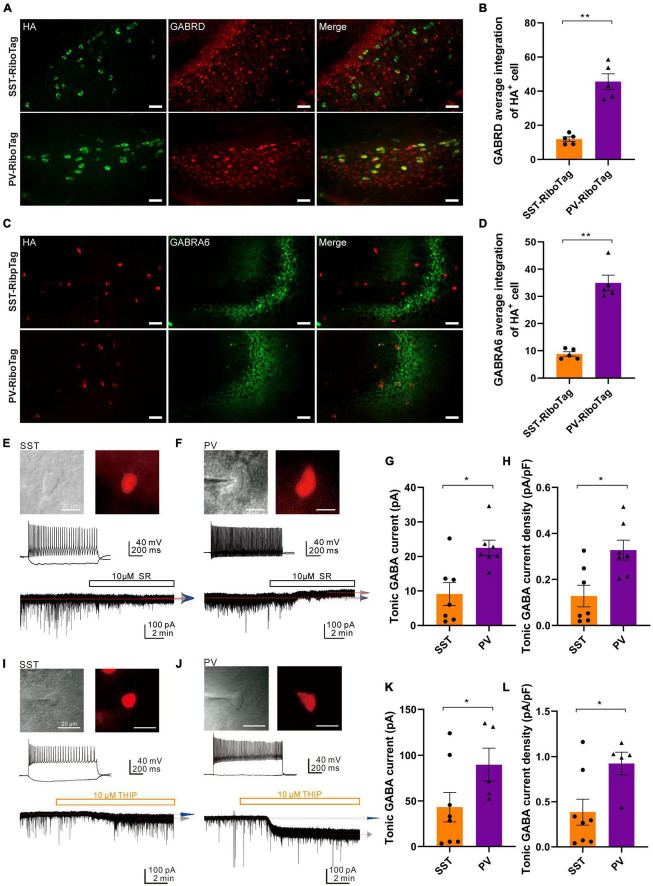
GABA_A_R subunits (GABRD and GABRA6) expression and tonic GABA_A_R-mediated currents in the SST and PV interneurons. **(A)** The co-immunofluorescence staining results of HA (green, left panel) and GABRD (red, middle panel) expression and the overlay images (right panel) in the hippocampal DG from SST-RiboTag (upper panel) and PV-RiboTag (lower panel) mice. Scale bar, 50 μm. **(B)** Quantification of GABRD average integration within HA-positive cells in the hippocampus. *n* = 5 SST-RiboTag mice (3-month-old, 3 males, 2 females); *n* = 5 PV-RiboTag mice (3-month-old, 3 males, 2 females). **p < 0.01. The GABRD-HA integration ratios were quantified and calculated by MetaMorph Premier analysis software; each dot in the graph represented the average results of 120 HA + cells from 1 mouse. Data are presented as the mean ± SEM and analyzed by the Mann–Whitney test. **(C)** The co-immunofluorescence staining results of HA (red, left panel) and GABRA6 (green, middle panel) expression and the overlay images (right panel) in the hippocampus from SST-RiboTag (upper panel) and PV-RiboTag (lower panel) mice. Scale bar, 50 μm. **(D)** Quantification of GABRA6 average integration within HA-positive cells in the hippocampus. *n* = 5 SST-RiboTag mice (3-month-old, 3 males, 2 females); *n* = 5 PV-RiboTag mice (3-month-old, 3 males, 2 females). **p < 0.01. The GABRA6-HA integration ratios were quantified and calculated by MetaMorph Premier analysis software; each dot in the graph represented the average results of 120 HA-positive cells from 1 mouse. Data are presented as the mean ± SEM and analyzed by the Mann–Whitney test. **(E)** IR-DIC and epifluorescence images showing an SST interneuron in the DG of an SST-Cre; Ai14 mouse brain. Scale bar, 20 μm. (top). The non-fast-spiking firing pattern of an SST interneuron (middle). Representative trace showing small tonic GABA_A_R-mediated current in the same neuron (*V*_hold_ = –70 mV) blocked by 10 μM SR95531 in the presence of 2 mM Kyn (bottom). **(F)** IR-DIC and epifluorescence images showing a PV interneuron in the DG of a PV-Cre; Ai14 mouse brain. Scale bar, 20 μm (top). The typical fast-spiking firing pattern of the PV interneuron (middle). Representative trace showing tonic GABA_A_R-mediated current in the neuron (*V*_hold_ = –70 mV) blocked by 10 μM SR95531 in the presence of 2 mM Kyn. Red dashed lines indicate Gaussian means (bottom). **(G)** Summary plot of the tonic GABA current obtained from SST- and PV-expressing interneurons in the DG. SST cells; *n* = 7 from 5 mice; PV cells, *n* = 7 from 5 mice; Mann–Whitney test, *U* = 6.0, *p* = 0.0175. Data are presented as the mean ± SEM. **(H)** Summary plot of the tonic current density (pA/pF) obtained from SST and PV interneurons in the DG. SST cells: *n* = 7 from 5 mice; PV cells: *n* = 7 from 5 mice. Mann-Whitney test, *U* = 7.0, *p = 0.0262. Data are presented as the mean ± SEM. **(I)** IR-DIC and epifluorescence images showing an SST interneuron in the DG of an SST-Cre; Ai14 mouse brain. Scale bar, 20 μm (top). The non-fast-spiking firing pattern of an SST interneuron (middle). Representative trace showing tonic δ-GABA_A_R-mediated current in the SST cell (*V*_hold_ = –70 mV) induced by 10 μM THIP in the presence of 2 mM Kyn. Red dashed lines indicate Gaussian means (bottom). **(J)** IR-DIC and epifluorescence images showing a PV interneuron in the DG of a mCherry-expressing PV-Cre mouse brain. Scale bar, 20 μm (top). The typical fast-spiking firing pattern of the PV interneuron (middle). Representative trace showing tonic δ-GABA_A_R-mediated current in the PV cell (*V*_hold_ = –70 mV) induced by 10 μM THIP in the presence of 2 mM Kyn. Red dashed lines indicate Gaussian means (bottom). **(K)** Summary plot of the tonic GABA current (pA) obtained from SST and PV interneurons in the DG (SST cells: 43.1 ± 16.1 pA, *n* = 8 cells from 2 mice; PV cells: 89.7 ± 17.9 pA, *n* = 5 cells from 3 mice; Mann–Whitney test, *U* = 6, *p = 0.0451). **(L)** Summary plot of the tonic current density (pA/pF) obtained from SST and PV interneurons in the DG (SST cells: 0.39 ± 0.14 pA/pF, *n* = 8 cells from 2 mice; PV cells: 0.92 ± 0.13 pA/pF *n* = 5 from 3 mice; Mann–Whitney test, *U* = 6, *p = 0.0451).

To confirm that the protein levels of the GABA_A_ receptor subunit GABRD and GABRA6 were higher in hippocampal PV interneurons, immunofluorescence co-staining against HA and GABRD was performed. The HA signal in green was used to represent the PV or SST interneurons in PV-RiboTag or SST-RiboTag mice, while the GABRD signal was depicted in red (Figure 5A). The average fluorescence intensity of GABRD in PV interneurons was significantly higher than that in SST interneurons (Figure 5B). Similarly, double staining GABRA6 (green) with HA (red) showed that GABRA6 is co-localized more with PV interneurons than with SST interneurons (Figures 5C, D). Taking the above results together, the relative GABRD and GABRA6 protein levels in PV or SST interneurons are consistent with mRNA findings using the RiboTag approach. The *Gabrd* gene encodes the δ-subunit of GABA_A_ receptors associated with extrasynaptic activity, which mediates tonic inhibition in CA1 and CA3 pyramidal cells, DG granule cells, and molecular layer interneurons (Glykys et al., 2008). While GABRA6 is expressed predominantly in cerebellar granule cells, the function of GABRA6 in the hippocampus is currently unknown. Previous studies had demonstrated that the δ subunit partners principally with the α6 subunit, δ and α6 subunits co-assemble and are necessary for tonic inhibition (Santhakumar et al., 2006). Therefore, the differential expression of GABRD and GABRA6 might contribute to the difference in tonic inhibitory currents between hippocampal PV and SST interneurons.

To determine the tonic GABA_A_R-mediated currents in these two types of interneurons, whole-cell recordings were made on SST and PV interneurons identified using SST-Cre; Ai14 and PV-Cre; Ai14 reporter mice, respectively (Figures 5E, F). The recordings were made in the presence of an ionotropic glutamate receptor blocker (2 mM Kyn). After recording inhibitory currents at the basal level, a GABA_A_R antagonist (10 μM SR95531) was added to abolish the phasic GABA_A_R-mediated sIPSCs and tonic (or sustained) GABA_A_R-mediated currents. The degree of inhibition of tonic GABA_A_R-mediated current was quantified by the shift of the baseline (Figures 5E, F, traces). Compared to the SST interneurons, the PV interneurons showed a prominent shift in the baseline current (Figures 5E, F, traces; SST: 68 ± 12.8 pA to 59 ± 10.4 pA; *n* = 7 cells; PV: 45 ± 9.0 pA to 22.5 ± 8.5 pA; *n* = 7 cells). Overall, the magnitude of tonic GABA currents recorded from the PV interneurons was significantly larger than that of SST interneurons (Figure 5G, PV interneurons, 22.5 ± 2.3 pA, *n* = 7 cells, 5 mice; SST interneurons, 9.1 ± 2.3 pA; *n* = 7 cells, 5 mice; Mann-Whitney test; *p* = 0.0175, *U* = 6.0). Moreover, the tonic current density was also significantly larger in PV interneurons (0.33 ± 0.04 pA/pF) than in SST interneurons (0.14 ± 0.05 pA/pF) (Figure 5H, Mann-Whitney test; *p* = 0.0262, *U* = 7.0).

To further compare the tonic δ-GABA_A_R-mediated currents between SST and PV interneurons, whole-cell patch-clamp recordings were made on SST and PV interneurons (Figures 5I, J) in the presence of an ionotropic glutamate receptor blocker (2 mM; Kyn). After recording basal level inhibitory currents, a δ-GABA_A_R agonist (10 μM; THIP) was applied to induce the δ-GABA_A_R-mediated tonic inhibitory currents. The degree of enhancement of δ-GABA_A_R-mediated tonic inhibitory current was quantified by the shift of the baseline (Figures 5I, J, traces). Compared to the SST interneurons, the PV interneurons showed a prominent shift in the baseline current (Figures 5I, J, traces; SST: −56.0 ± 6.4 pA to −99.1 ± 12.1 pA, *n* = 8 cells; PV: −12.2 ± 4.1 pA to −102.0 ± 12.1 pA, *n* = 5 cells). Overall, the magnitude of δ-GABA_A_R-mediated tonic inhibitory currents recorded from the PV interneurons was significantly larger than that of SST interneurons (Figure 5K; SST: 43.1 ± 16.1 pA, *n* = 8 cells from 2 mice; PV: 89.7 ± 17.9 pA, *n* = 5 cells from 3 mice; Mann-Whitney test, *U* = 6, *p* = 0.0451). Moreover, the tonic inhibitory current density was also significantly larger in PV interneurons than in SST interneurons (Figure 5L, SST: 0.39 ± 0.14 pA/pF, *n* = 8 cells from 2 mice; PV: 0.92 ± 0.13 pA/pF *n* = 5 from 3 mice; Mann-Whitney test, *U* = 6, *p* = 0.0451). Collectively, the result demonstrated that PV interneurons exhibit larger δ-GABA_A_R-mediated tonic inhibitory currents than SST interneurons in the hippocampal DG.

### 3.6. DEGs between PV and SST interneurons are associated with schizophrenia and epilepsy

**FIGURE 6:**
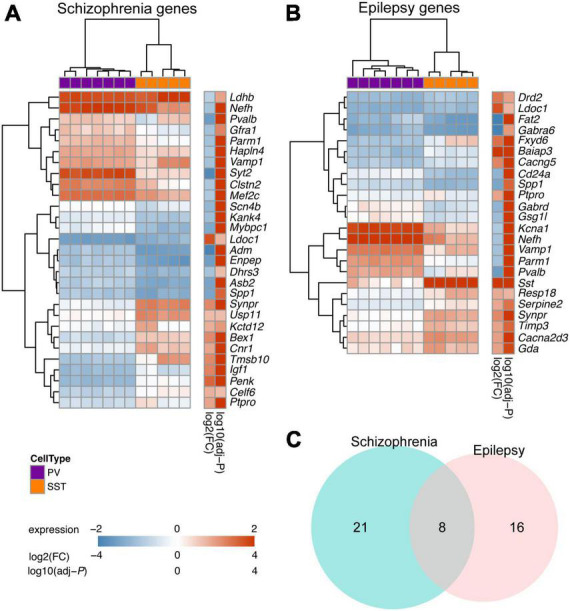
Overlapping DEGs between PV and SST interneurons with disease-related genes for schizophrenia and epilepsy. **(A)** Heatmap showing the top DEGs between SST and PV interneurons (adjusted-*P* < 10^–5^ in Enrichr enrichment analysis) in schizophrenia-associated gene sets. **(B)** Top DEGs between SST and PV interneurons (adjusted-*P* < 0.05 in Enrichr enrichment analysis) in epilepsy-associated gene sets. For panels **(A,B)**, the Color bar shows variance stabilizing transformed expression, log2(fold change), and log10(adjusted *P*). **(C)** Venn diagram showing overlap of DEGs within schizophrenia-associated gene sets and epilepsy-associated gene sets.

Finally, going beyond their physiological functions, we delved into exploring potential disease-associated DEGs between PV and SST interneurons. Dysfunction of PV or SST interneurons has been implicated in the development of schizophrenia and temporal lobe epilepsy (Tallent and Qiu, 2008; Drexel et al., 2017). Our study revealed a strong association between the DEGs of PV and SST interneurons and pathways related to schizophrenia. Specifically, the analysis identified 29 DEGs between PV and SST interneurons that were involved in schizophrenia-related pathways (Figure 6A). In addition, 24 DEGs between PV and SST interneurons overlapped with epilepsy-related pathways (Figure 6B). There were 8 DEGs (*Ldoc1*, *Nefh*, *Parm1*, *Ptpro*, *Pvalb*, *Spp1*, *Synpr*, *Vamp1*) shared by the schizophrenia and epilepsy gene sets (Figure 6C), suggesting a common molecular basis between the two disorders.

## 4. Discussion

### 4.1. Main findings

The molecular mechanisms underlying the specialized properties of PV and SST interneurons are yet to be clarified to date. In this study, SST interneurons exhibited increased expression of various voltage-gated Ca^2+^ channels, whereas PV interneurons showed heightened expression of Na^+^ and K^+^ channels. In addition, both mRNA and protein levels of the GABA_A_ receptor δ and α6 subunits were higher in the PV interneurons than in SST interneurons. These two subunits of the GABA_A_ receptor are predominantly found extrasynaptically and contribute to tonic inhibition (Santhakumar et al., 2006; Tong et al., 2015), in agreement with the larger tonic currents recorded in PV interneurons than those in SST interneurons. Compared to gain control by phasic inhibition, tonic inhibition of PV interneurons can offset their output in response to input (Mitchell and Silver, 2003). Finally, the transcriptomic analysis revealed numerous DEGs associated with schizophrenia and epilepsy, providing a foundation for future studies of these genes.

### 4.2. Comparison of RiboTag and other methods

Specific cell-type transcriptomics is primarily conducted using single-cell sequencing, which isolates mRNA from single cells using droplet microfluidic-based technology (Hwang et al., 2018) or the patch-seq approach (Lipovsek et al., 2021). The advantages of microfluidic technology are high throughput, small sample volume, and low cross-contamination (Brouzes et al., 2009; Hwang et al., 2018). However, isolating the cells from the physiological tissue context requires mechanical processing and enzymatic digestion, which increases the risk of artifacts and introduces a bias toward subpopulations (Halldorsson et al., 2015; Haimon et al., 2018). The sequencing data from cell types with complicated morphology and low abundance may be harder to be acquired. Patch-seq collects mRNA after electrophysiological recordings and morphological reconstruction of the same cells. The major advantage of this technique is that it enables the study of the transcriptome, anatomical position, electrical properties, and morphological structure of a single neuron. However, a main limitation is a very low throughput due to the requirement of interdisciplinary skills that take years to master (Lipovsek et al., 2021). Another limitation is the possibility of contamination because the pipette may pick up debris RNA from the extracellular space while reaching the target cell (Cadwell et al., 2017).

The RiboTag method allows us to robustly collect mRNA from specific subtypes of interneurons in tissue extracts. Compared with other approaches, the RiboTag method is more appropriate for simultaneously comparing transcriptomes of one specific cell type in multiple mice after behavioral tests or drug treatment. Furthermore, comparing the mRNA between whole tissue (input) and specific cell type (IP) from the same mouse provides baseline information on the experimental animal (Lesiak et al., 2015). However, one of the limitations of the RiboTag method is its inability to detect non-coding RNAs (ncRNAs), which do not bind to ribosomes. Two types of ncRNAs, micro RNAs, and long non-coding RNAs, have been implicated in regulating brain development, homeostasis, neurodegeneration, and plasticity. For example, the Evf-2 lncRNA is vital for GABAergic neuron development by regulating gene expression (Wei et al., 2018). Another limitation of the RiboTag method is the lack of information on the sub-population of interneurons. Interneurons expressing the same molecular marker may have different electrophysiological properties (Markram et al., 2015; Tremblay et al., 2016; Zeng and Sanes, 2017). Given that this approach collects all the mRNA from cell expression of the same Cre recombinase, information on the interneuron subtype cannot be acquired.

### 4.3. The comparison and limitation of this study

The PV and SST interneurons follow a similar developmental organization in the hippocampus and cortex (Yao et al., 2021). To determine the regional similarities or differences in gene expression in PV and SST interneurons, we compared the DEGs in our study to those identified in the mouse cortex (Huntley et al., 2020). The overlapping up- and down-regulated genes in the two studies were provided in Supplementary Table 9. Notably, *Reln*, a gene that generally co-expressed with SST interneurons (Pohlkamp et al., 2014), was significantly up-regulated in SST interneurons in both the mouse hippocampus and cortex. On the other hand, *Syt2* was significantly up-regulated in PV interneurons in the hippocampus and cortex. Several ion channel genes were also expressed in the cortex, such as *Cacna2d3*, *Cacng4*, and *Cacng5* in SST interneurons, and *Kcng4* and *Kcnc3* in PV interneurons. However, *Scn4b*, *Kcnab1*, and *Kcna1* were not up-regulated in PV interneurons in the cortex.

Although *Sst* and *Pvalb* have been utilized as markers for SST and PV interneurons, respectively, it is worth noting that their expression is not exclusive to their respective cell types. SST or PV interneurons can be further divided into subtypes using single-cell RNA-seq. In the mouse cortex and hippocampus, a small fraction of SST interneurons expresses *Pvalb*, while a small fraction of PV interneurons expresses *Sst* (Gouwens et al., 2020; Yao et al., 2021). This co-expression is also reported in Allen Brain Map Transcriptomics Explorer (modified in Supplementary Figure 1A). Indeed, our data revealed a low level of *Pvalb* expression in SST interneurons (Figure 1E; Supplementary Figure 1B), suggesting that a subtype of *Pvalb*-expressing SST interneurons was immunoprecipitated with the RiboTag approach. The co-expression could be a confounding factor to our transcriptome analysis. However, it is important to acknowledge that the PV-Cre and SST-Cre lines employed in this study are widely used in various interneuron investigations (Zou et al., 2016; Drexel et al., 2017; Gouwens et al., 2020; Jang et al., 2020; Udakis et al., 2020; Morales et al., 2021; Asgarihafshejani et al., 2022; Drexel et al., 2022). Therefore, our findings can offer valuable molecular insights for the utilization of these lines.

This study has additional limitations, as it does not account for the transcriptomic variations between PV and SST interneurons in specific hippocampal subregions, such as CA1 and DG, where these neurons may display distinct transcriptomic profiles in response to various conditions like diseases or behavioral paradigms. PV and SST interneurons show different morphological, molecular, and electrophysiological properties across the hippocampal subregions (Botcher et al., 2014). Furthermore, PV and SST interneurons are involved in different memory types. For example, CA1 PV interneurons are crucial for spatial working memory (Murray et al., 2011), whereas CA1 SST interneurons control contextual fear memory and regulate object location memory (Lovett-Barron et al., 2014; Asgarihafshejani et al., 2022; Honore and Lacaille, 2022). DG PV interneurons modulate anxiety, social interaction, and memory extinction (Zou et al., 2016); nevertheless, DG SST interneurons are required for contextual and spatial overlapping memories (Morales et al., 2021). In the future, it is imperative to consider the hippocampal subregional heterogeneity of PV and SST interneurons to investigate their role in neuropsychiatric disorders or learning and memory.

### 4.4. Dysregulation of interneuron gene expression in schizophrenia and epilepsy

Schizophrenia and epilepsy are two brain disorders associated with the dysfunction of interneurons. In schizophrenia, the impaired excitatory-inhibitory (E-I) balance and reduced oscillatory activity result from decreased GABAergic signaling due to hypoexcitability of PV interneurons (Lodge et al., 2009; Lewis et al., 2012). Similarly, various forms of epilepsy, such as temporal lobe epilepsy, SCN8A epileptic encephalopathy, and Dravet syndrome, are associated with an imbalanced E-I ratio arising from dysfunction in SST and PV interneurons (Tai et al., 2014; Wengert et al., 2021; Drexel et al., 2022). Although the pathophysiology mechanisms of these neuropsychiatric disorders are not clearly understood, insights into the gene expression profiles of PV or SST interneurons can provide valuable indications.

Between SST and PV transcriptomes, we identified 29 and 24 DEGs overlapping with gene sets involving schizophrenia and epilepsy, respectively. The voltage-gated sodium channel β subunit 4 (*Scn4b*) is expressed higher in PV interneuron and serves as a marker in the dorsolateral prefrontal cortex for schizophrenia (Guillozet-Bongaarts et al., 2014). Schizophrenia is associated with reduced excitability of PV interneurons. It is of interest to investigate the potential correlation between the higher *Scn4b* expression and the hypoexcitability in PV interneurons, and how they underlie the schizophrenia phenotype. Furthermore, *Cacna2d3*, a gene encoding voltage-dependent calcium channel subunit α2/δ3, has been implicated as a candidate gene for epilepsy (Peng et al., 2021). Besides, impairment in dendritic inhibition, rather than somatic inhibition, has been observed in various in several forms of epilepsy (Cossart et al., 2001; Wittner et al., 2001; Wittner et al., 2005; Sanjay et al., 2015). SST interneurons play a role in dendritic inhibition, contributing to the regulation of Ca^2+^ signaling, while PV interneurons primarily regulate hippocampal network oscillations through perisomatic inhibition (Udakis et al., 2020). Future studies will be required to test if the reduced expression of *Cacna2d3* in SST interneurons leads to decreased dendritic inhibition, which in turn to epileptic features. Moreover, we detected eight genes associated with both schizophrenia and epilepsy that were differentially expressed between SST and PV interneurons, thus providing further direction for their roles in these diseases.

This article highlights *Gabrd*, a gene implicated in both schizophrenia and epilepsy. The link between differential tonic inhibition in SST and PV interneurons suggests that the GABA_A_ receptor δ subunit may be a possible target for schizophrenia/epilepsy drugs. Another GABA_A_ receptor examined in our study, *Gabra6*, is predominantly expressed in the cerebellum under physiological conditions. In the hippocampus, it is associated with stress-induced depressive behaviors (Yang et al., 2016). Mutations in *Gabra6* have also been identified in patients with idiopathic generalized epilepsy (Riaz et al., 2021), and its single-nucleotide polymorphism is associated with schizophrenia (Gong et al., 2013). In our study, the mRNA and protein level of *Gabra6* was higher in hippocampal PV interneurons than in SST interneurons under physiological conditions. Because our experiments were conducted under normal physiological conditions, further studies using disease models are required to confirm the relationship between these gene expressions and disease development.

In conclusion, this study demonstrates the feasibility of employing the RiboTag-seq approach for investigating gene expression profiles specific to different cell types. The results reveal the molecular signatures that may be involved in the physiological functions of particular interneuron types and provide valuable insights into their implications in brain disorders.

## Data availability statement

The datasets presented in this study are deposited in the online repositories. The names of the repository/repositories and accession number(s) can be found below: https://dataverse.lib.nycu.edu.tw/dataset.xhtml?persistentId=doi:10.57770/U1BJQ8, NYCU Dataverse.

## Ethics statement

The animal study was reviewed and approved by the Institutional Animal Care and Use Committee (IACUC) of the National Yang Ming Chiao Tung University.

## Author contributions

C-YC, C-CL, C-DC, and IC contributed to the conception and design of the study, edited, and revised the manuscript. T-HH, C-WH, and MA performed the experiments. Y-SL, L-YW, and C-YC analyzed the RNA-seq data. T-HH, Y-SL, and C-WH wrote the first draft of the manuscript. All authors read and approved the submitted version.

## Footnotes

1
https://github.com/kevinblighe/EnhancedVolcano

## Supplementary material

Supplementary Table 1 Chemicals and products list.

Supplementary Table 2 Antibodies used in this study.

Supplementary Table 3 List of mice information in this study.

Supplementary Table 4 Primer list for RT-qPCR.

Supplementary Table 5 List of differentially expressed genes (DEGs) between SST and PV interneurons.

Supplementary Table 6 List of enriched GO terms for up-regulated genes in SST interneurons.

Supplementary Table 7 List of enriched GO terms for up-regulated genes in PV interneurons.

Supplementary Table 8 List of enriched KEGG pathways for DEGs between SST and PV interneurons.

Supplementary Table 9 List of shared DEGs in this study and Huntley et al., 2020.
