# Supplementary figures and images for: Differential expression of GABAA receptor subunits δ and α6 mediates tonic inhibition in parvalbumin and somatostatin interneurons in the mouse hippocampus
Source: Front Cell Neurosci. 2023 Jul 20;17:1146278. doi: 10.3389/fncel.2023.1146278 (PMC10397515; doi:10.3389/fncel.2023.1146278)

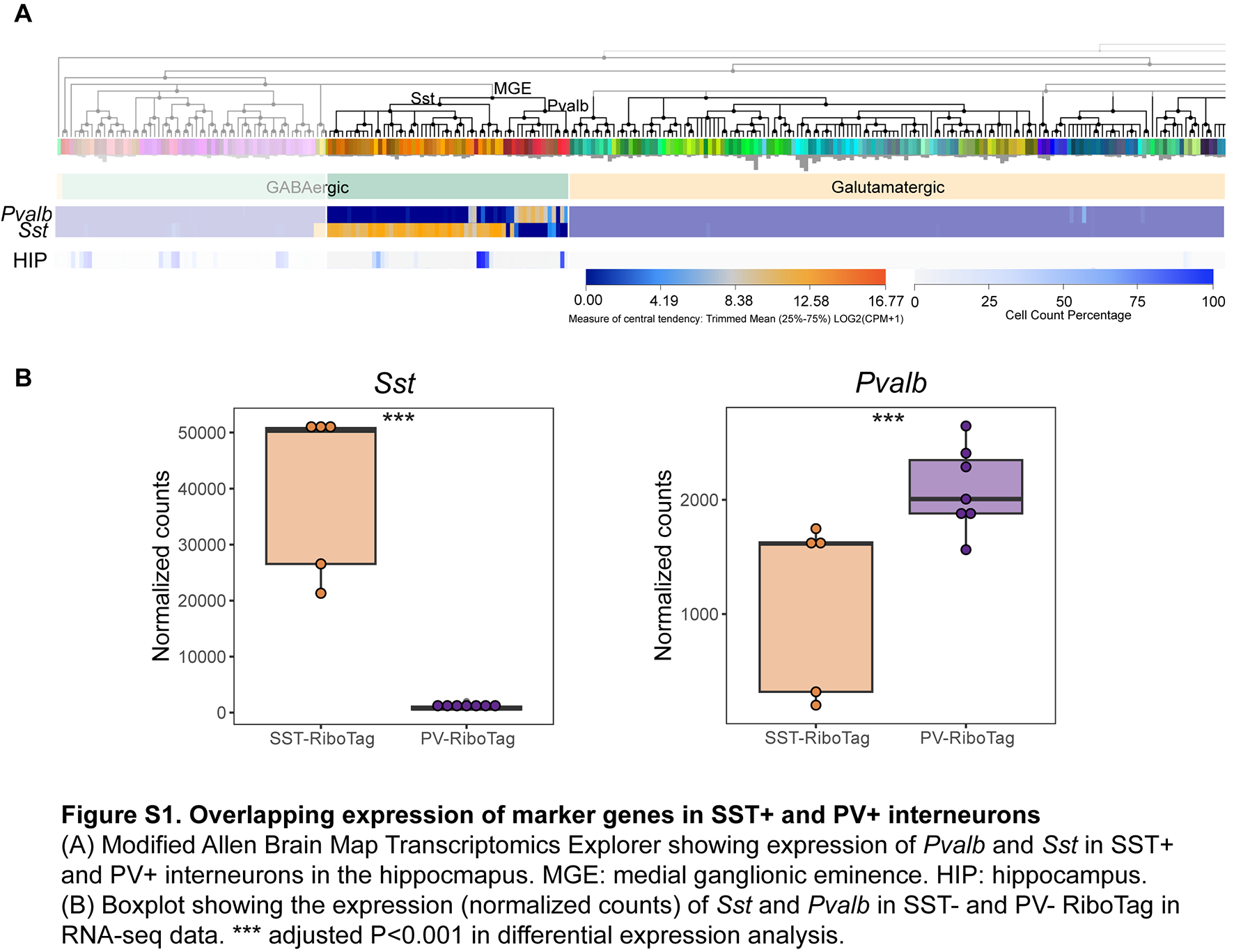

Supplement: Supplementary file 1 [file Image_1.TIF]
