# Supplementary material for: Differential expression of GABAA receptor subunits δ and α6 mediates tonic inhibition in parvalbumin and somatostatin interneurons in the mouse hippocampus
Source: Front Cell Neurosci. 2023 Jul 20;17:1146278. doi: 10.3389/fncel.2023.1146278 (PMC10397515; doi:10.3389/fncel.2023.1146278)
Supplement: Supplementary Table 1 — Chemicals and products list. [file Table_1.pdf]

Supplementary Table 1. Chemicals and products list.

Experiment	Product	Supplier	Catalog No.
mRNA extraction from HA-tagged ribosomes	Trizma® hydrochloride solution	Sigma-Aldrich	T2663
	$MgCl_2$ (1M)	Thermo Fisher Scientific	AM9530G
	KCl (2M)	Thermo Fisher Scientific	AM9640G
	Dithiothreitol (DTT)	Omics Bio	DB0058
	NONIDET P-40 SUBSTITUTE	BioShop Canada Inc.	NON505
	Recombinant RNasin® Ribonuclease Inhibitor	Promega Corporation	N251B
	Heparin	Sigma-Aldrich	H3393
	cOmplete™, EDTA-free Protease Inhibitor Cocktail	Roche	4693132001
	UltraPure™ DNase/RNase-Free Distilled Water	Thermo Fisher Scientific	10977015
	Dynabeads™ Protein G for Immunoprecipitation	Thermo Fisher Scientific	10003D
	Citric acid	Thermo Fisher Scientific	251275
	Sodium Phosphate, Dibasic	Millipore	567547
	Ethanol	Bioman Scientific	E23
	2-Mercaptoethanol	Sigma-Aldrich	M7154
	RNeasy Micro Kit	QIAGEN	74004
PCR and RT-qPCR	2X PCR Dye Master Mix	ARROWTEC	ADPMX02D-100
	2X PCRBIO VeriFi Mix Red	PCR Biosystems	SA-PB10.44-5
	2X qPCRBIO SyGreen Blue Mix HI-ROX	PCR Biosystems	PB20.16-01
Immuno-histochemistry	Paraformaldehyde	Sigma-Aldrich	P6148
	Sucrose	Bio Basic Inc.	SB0498
	Tissue-Tek® O.C.T. Compound	Sakura Finetek USA, Inc.	4583
	10X PBS buffer, pH7.4	Omics Bio	IB3012
	Triton X-100	Bio Basic Inc.	DB0198
	FBS	NQBB	A6806-11
	Bovine Serum Albumin (BSA) Fraction V	Bio Basic Inc.	AD0023
	Sodium azide	Honeywell Riedel-de Haen	13412
	Antifade Mounting Medium with DAPI	Vector Laboratories.	H-1200
