# Supplementary material for: Differential expression of GABAA receptor subunits δ and α6 mediates tonic inhibition in parvalbumin and somatostatin interneurons in the mouse hippocampus
Source: Front Cell Neurosci. 2023 Jul 20;17:1146278. doi: 10.3389/fncel.2023.1146278 (PMC10397515; doi:10.3389/fncel.2023.1146278)
Supplement: Supplementary Table 2 — Antibodies used in this study. [file Table_2.pdf]

Supplementary Table 2. Antibodies used in this study.

Experiment	Immunogen	Host species	Supplier	Catalog No.	Dilution
RiboTag immunoprecipitation	HA tag	Rabbit Polyclonal	Abcam	ab9110	1:50
Immunoblotting (Primary antibody)	HA tag	Rabbit Polyclonal	Abcam	ab9110	1:1000
	beta III Tubulin	Mouse Monoclonal	Abcam	ab7751	1:2000
Immunoblotting (Secondary antibody)	HRP-conjugated Anti-Mouse IgG	Goat Polyclonal	Jackson Immuno-Research	115-035-003	1:1000
	HRP-conjugated Anti-Rabbit IgG	Mouse Monoclonal	Rockland	18-8816-31	1:1000
Immunofluorescence staining (Primary antibody)	HA tag	Rabbit Polyclonal	GeneTex	GTX115044	1:200
	HA.11 Epitope Tag	Mouse Monoclonal	BioLegend	MMS-101R	1:200
	Somatostatin	Rat Monoclonal	Millipore	MAB354	1:100
	Parvalbumin	Mouse Monoclonal	Millipore	MAB1572	1:1000
	GABRD (extracellular)	Rabbit Polyclonal	Invitrogen	PA5-77408	1:200
	GABA-AR alpha 6	Rabbit Polyclonal	Novus Biologicals	NB300-196	1:200
Immunofluorescence staining (Secondary antibody)	Alexa Fluor 488 anti-rabbit IgG	Goat Polyclonal	Invitrogen	A11008	1:500
	Alexa Fluor 594 anti-rabbit IgG	Goat Polyclonal	Invitrogen	A11012	1:500
	Alexa Fluor 488 anti-mouse IgG	Goat Polyclonal	Invitrogen	A11001	1:500
	Alexa Fluor 594 anti-rat IgG	Goat Polyclonal	Invitrogen	A11007	1:1000
