# Supplementary material for: Differential expression of GABAA receptor subunits δ and α6 mediates tonic inhibition in parvalbumin and somatostatin interneurons in the mouse hippocampus
Source: Front Cell Neurosci. 2023 Jul 20;17:1146278. doi: 10.3389/fncel.2023.1146278 (PMC10397515; doi:10.3389/fncel.2023.1146278)
Supplement: Supplementary Table 3 — List of mice information in this study. [file Table_3.pdf]

Supplementary Table 3. List of mice information in this study.

Figure/ Experiment	Age	Number/ Gender	Genotype	Statistical analysis
1E<br>*Gfap*<br>RT-qPCR	3 mon	6 females	SST-RiboTag	Mann-Whitney test, two-tailed<br>*p* value=0.0649<br>Actual Difference:-0.02608
	3 mon	6 females	PV-RiboTag	
1E<br>*Cnp1*<br>RT-qPCR	3 mon	7 females	SST-RiboTag	Mann-Whitney test, two-tailed<br>*p* value=0.7922<br>Actual Difference:-0.004657
	3 mon	1 male<br>6 females	PV-RiboTag	
1E<br>*Iba1*<br>RT-qPCR	3 mon	6 females	SST-RiboTag	Mann-Whitney test, two-tailed<br>*p* value=0.5887<br>Actual Difference:0.03880
	3 mon	3 males<br>3 females	PV-RiboTag	
1E<br>*Camk2a*<br>RT-qPCR	3 mon	6 females	SST-RiboTag	Mann-Whitney test, two-tailed<br>*p* value=0.1508<br>Actual Difference:-0.01952
	3 mon	5 females	PV-RiboTag	
1E<br>*Gad1*<br>RT-qPCR	3 mon	1 male<br>6 females	SST-RiboTag	Mann-Whitney test, two-tailed<br>*p* value=0.0260<br>Actual Difference:2.538
	3 mon	6 females	PV-RiboTag	
1E<br>*Pvalb*<br>RT-qPCR	3 mon	1 male<br>9 females	SST-RiboTag	Mann-Whitney test, two-tailed<br>*p* value=0.0002<br>Actual Difference:20.70
	3 mon	6 females	PV-RiboTag	
1E<br>*Sst*<br>RT-qPCR	3 mon	1 male<br>10 females	SST-RiboTag	Mann-Whitney test, two-tailed<br>*p* value=0.0002<br>Actual Difference:-20.03
	3 mon	6 females	PV-RiboTag	
2BCDEF, 4BC, 6ABC<br>RNAseq and bioinformatic analysis	3 mon	5 males	SST-RiboTag	Please see supplement table 5-9 for more detail
	3 mon	7 males	PV-RiboTag	
2G<br>*Cacna2d3*<br>RT-qPCR	3 mon	2 males<br>4 females	SST-RiboTag	Mann-Whitney test, two-tailed<br>*p* value=0.0016<br>Actual Difference:-0.4086
	3 mon	6 males<br>3 females	PV-RiboTag	
2G<br>*Scn4b*<br>RT-qPCR	3 mon	2 males<br>5 females	SST-RiboTag	Mann-Whitney test, two-tailed<br>*p* value=0.0003<br>Actual Difference:2.918
	3 mon	5 males<br>4 females	PV-RiboTag	
2G<br>*Kcnc3*<br>RT-qPCR	3 mon	2 males<br>5 females	SST-RiboTag	Mann-Whitney test, two-tailed<br>*p* value=0.0006<br>Actual Difference:0.9511
	3 mon	4 males<br>3 females	PV-RiboTag	

2G<br>*Kcng4*<br>RT-qPCR	3 mon	2 males<br>4 females	SST-RiboTag	Mann-Whitney test, two-tailed<br>*p* value=0.0012<br>Actual Difference:3.411
	3 mon	4 males<br>3 females	PV-RiboTag	
2G<br>*Grin3a*<br>RT-qPCR	3 mon	3 males<br>4 females	SST-RiboTag	Mann-Whitney test, two-tailed<br>*p* value=0.0012<br>Actual Difference:-0.9946
	3 mon	3 males<br>3 females	PV-RiboTag	
4D<br>*Gabrd*<br>RT-qPCR	3 mon	6 females	SST-RiboTag	Mann-Whitney test, two-tailed<br>*p* value=0.0022<br>Actual Difference:2.175
	3 mon	1 male<br>5 females	PV-RiboTag	
4D<br>*Gabra6*<br>RT-qPCR	3 mon	6 females	SST-RiboTag	Mann-Whitney test, two-tailed<br>*p* value=0.0043<br>Actual Difference:9.562
	3 mon	1 male<br>5 females	PV-RiboTag	
5B<br>immunofluorescence images	3 mon	3 males<br>2 females	SST-RiboTag	Mann-Whitney test, two-tailed<br>*p* value=0.0079<br>Actual difference:30.82
	3 mon	2 males<br>3 females	PV-RiboTag	
5D<br>immunofluorescence images	3 mon	3 males<br>2 females	SST-RiboTag	Mann-Whitney test, two-tailed<br>*p* value=0.0079<br>Actual Difference:24.40
	3 mon	2 males<br>3 females	PV-RiboTag	
5G<br>Tonic GABA current	2-3 mon	3 males<br>2 females	SST-Cre; Ai14	Mean-Whitney test, two-tailed<br>*p* value=0.0175<br>Actual Difference:14.67
	2-3 mon	3 males<br>2 females	PV-Cre; Ai14	
5H<br>Tonic GABA current density	2-3 mon	3 males<br>2 females	SST-Cre; Ai14	Mann-Whitney test, two-tailed<br>*p* value=0.0262<br>Actual Difference:0.2498
	2-3 mon	3 males<br>2 females	PV-Cre; Ai14	
5K<br>Tonic GABA current	4-6 mon	1 male<br>1 female	SST-Cre; Ai14	Mann-Whitney test, two-tailed<br>*p* value=0.0451<br>Actual Difference: 41.10
	4-6 mon	2 males<br>1 female	PV-Cre; Virus<br>PV-Cre; Ai14	
5L<br>Tonic GABA current density	4-6 mon	1 male<br>1 female	SST-Cre; Ai14	Mann-Whitney test, two-tailed<br>*p* value=0.0451<br>Actual Difference: 0.7375
	4-6 mon	2 males<br>1 female	PV-Cre; Virus<br>PV-Cre; Ai14	
