# Supplementary material for: Differential expression of GABAA receptor subunits δ and α6 mediates tonic inhibition in parvalbumin and somatostatin interneurons in the mouse hippocampus
Source: Front Cell Neurosci. 2023 Jul 20;17:1146278. doi: 10.3389/fncel.2023.1146278 (PMC10397515; doi:10.3389/fncel.2023.1146278)
Supplement: Supplementary Table 4 — Primer list for RT-qPCR. [file Table_4.pdf]

**Supplementary Table 4**. Primer list for RT-qPCR.

| Gene name | Primer sequence (5'→3') | Gene accession number |
|---|---|---|
| *Cacna2d3* | Fwd: TGGTGGGGAGATAAAATCCA | NM_009785.1 |
| | Rev: CCTCGGACTTCTTGTGGAAC | |
| *Camk2a* | Fwd: TTTGAGGAACTGGGAAAGGG | NM_177407.4 |
| | Rev: CATGGAGTCGGACGATATTGG | |
| *Cnp1* | Fwd: GCTGCACTGTACAACCAAATTCTG | NM_001146318.1 |
| | Rev: ACCTCCTGCTGGGCGTATT | |
| *Gabra6* | Fwd: CTCTACCCAAAGTGTCCTATGC | NM_001359049.1 |
| | Rev: GTGACTGGAGATTGGTGAAGTAG | |
| *Gabrd* | Fwd: CCTTCATCGTGAATGCCAAATC | NM_008072.2 |
| | Rev: GGTGATGCGGATGCTGTATAA | |
| *Gad1* | Fwd: TTGTGCTTTGCTGTGTTTTAGAGA | NM_008077.5 |
| | Rev: CCCCCTGCCCAAAGATAGAC | |
| *Gapdh* | Fwd: GCATCCACTGGTGCTGCC | NM_001289726.1 |
| | Rev: TCATCATACTTGGCAGGTTTC | |
| *Gfap* | Fwd: AGCGAGCGTGCAGAGATGA | NM_001131020.1 |
| | Rev: AGGAAGCGGACCTTCTCGAT | |
| *Grin3a* | Fwd: TTGTCATGGACTCGGGAATATG | NM_001276355.1 |
| | Rev: TTCAATCAGTGTCACCACTCTC | |
| *Iba1* | Fwd: CCCCCAGCCAAGAAAGCTAT | NM_001361501.1 |
| | Rev: GCCCCACCGTGTGACATC | |
| *Kcnc3* | Fwd: CTAAGGTCGGAGCTAGCATTT | NM_008422.3 |
| | Rev: TCCTACGTGTTCCTAGAGGTT | |
| *Kcng4* | Fwd: CAGGCAGAGAGTGGTTCTATT | NM_025734.2 |
| | Rev: TGGATTCCACCACGAAGATG | |
| *Pvalb* | Fwd: TGTCGATGACAGACGTGCTC | NM_013645 (Filice et al. 2016) |
| | Rev: TTCTTCAACCCCAATCTTGC | |
| *Scn4b* | Fwd: CCACCAAGGAGAAGACGAATAA | NM_001013390.3 |
| | Rev: CCTCACGAAGCAGGTGTATC | |
| *Sst* | Fwd: GCCCAACCAGACAGAGAATGA | NM_009215.1 |
| | Rev: AAGTTCTTGCAGCCAGCTTTG | |
